# A peptide puzzle

**DOI:** 10.7554/eLife.41524

**Published:** 2018-12-07

**Authors:** Jian Guan, Nilabh Shastri

**Affiliations:** Department of PathologyJohns Hopkins School of MedicineBaltimoreUnited States

**Keywords:** tumor antigens, genetic heterogeneity, tumor evolution, tumor resistance to immune response, cancer immunology, cancer immunotherapy, Mouse

## Abstract

Why does cancer develop in situations where the immune system is perfectly capable of eliminating it?

**Related research article** Gejman RS, Chang AY, Jones HF, DiKun K, Hakimi AA, Schietinger A, Scheinberg DA. 2018. Rejection of immunogenic tumor clones is limited by clonal fraction. *eLife* **7**:e41090. doi: 10.7554/eLife.41090

The primary function of the immune system is to detect and eliminate any abnormal cells in the body. To detect these abnormal cells, immune cells called 'killer cells' look for changes in the peptides that are present on the surface of all cells. In healthy cells these peptides stand for normal proteins made inside the cell. However, if new peptides are found, the killer cells take that as evidence of abnormality, such as a virus infection or cancer, and they destroy the abnormal cells to limit the spread of an infection or the growth of a cancer. So why do the killer cells fail to prevent the growth of some tumors in the first place?

The idea that immune system could contain the growth of tumors has been controversial for many decades (Burnet, 1967; Hellström et al., 1968). However, the recent success of immunotherapy – which was highlighted when the 2018 Nobel Prize in Physiology or Medicine was awarded to James Allison and Tasuku Honjo – has dramatically improved the prospects of cancer treatment. Many, but not all, patients with previously incurable cancers have effectively been cured by immunotherapy.

Making further improvements, to help patients who are not responsive to immunotherapy at present, will require a better understanding of how the body regulates the response of killer cells to cancer. This is especially important during the early stages of cancer when there are relatively few abnormal cells. Now, in eLife, David Scheinberg and colleagues at Weill Cornell Medicine and Memorial Sloan Kettering Cancer Center – including Ron Gejman and Aaron Chang as joint first authors – report the results of studies in which an elegant new experimental platform called PresentER was used to study the response of killer cells to thousands of different peptides in mice (Gejman et al., 2018).

Cancer cells were injected into immunocompetent mice and left to grow for several weeks. Some cancer cells were detected and destroyed by the immune system, while others failed to be eliminated and grew into tumors. When Gejman et al. analyzed the cells in these tumors they found to their surprise that, in general, the presence of a particular peptide did not result in detection and rejection: this was also true even for immunogenic peptides (that is, for peptides that are known to elicit a strong response from the immune system). Rather, the tumors that developed tended to contain cells expressing a wide range of different immunogenic peptides. This suggests that the immune system can only detect and reject a tumor when a certain fraction of the cells in the tumor display the same immunogenic peptide. This behavior is particularly interesting because it is similar to what is seen in human cancer patients who do not benefit from immunotherapy (McGranahan et al., 2016).

Why does the immune system fail to reject cancer cells that display a heterogenous mix of peptides? To explore this question Gejman et al. injected mice with mixtures of cancer cells in which some of the cells displayed immunogenic peptide, while the rest displayed non-immunogenic peptides. When the fraction of cells with immunogenic peptides was low, the cells were not eliminated (Figure 1). Moreover, the minimum fraction required to generate an effective immune response varied between different peptides, suggesting that some as-yet-unknown features of the peptides were important.

**Figure 1.**
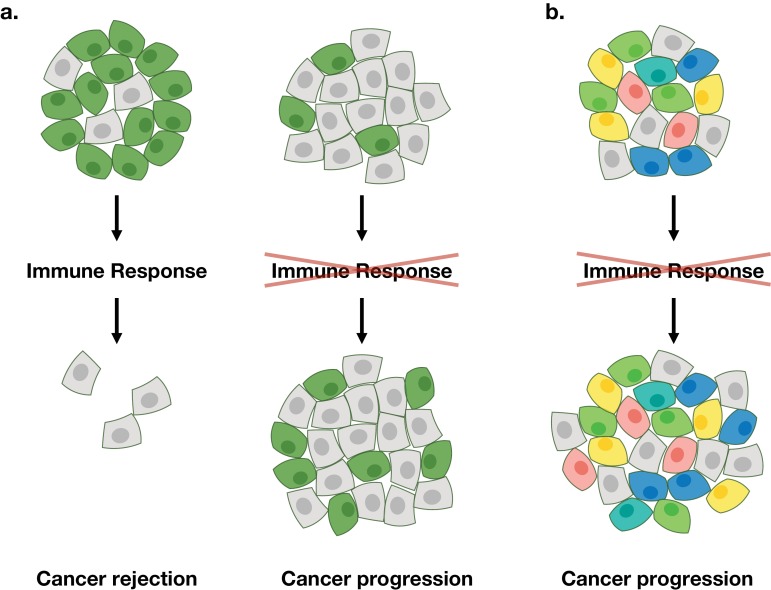
Schematic illustration of the anti-cancer immune response. (**a**) Gejman et al. found that a cancer that contains a large fraction of immunogenic tumor cells of the same type (shown in green) can be effectively rejected by the immune system of the mouse (left), whereas a cancer that contains a small fraction of immunogenic tumor cells will not be rejected (right). (**b**) However, a cancer that contains a large fraction of immunogenic tumor cells of different types (shown in different colors) will not be rejected. This behavior observed by Gejman et al. in mice is similar to that seen in humans who do not respond to immunotherapy (McGranahan et al., 2016).

The work of Gejman et al. establishes that peptide heterogeneity within cancer cells has an impact on the detection of cancer and also on the responsiveness to immunotherapy. It also highlights the influence of the fraction of the immunogenic cells in a given cancer, a factor that has largely been underestimated up until now, and the need for a better understanding of the role of immunogenic peptides in the generating an effective immune response to cancer. And last, but not least, this latest work shows the potential of the PresentER approach to be used in large-scale screening studies of potentially immunogenic peptides.
